# Bacterial Response to Oxidative Stress and RNA Oxidation

**DOI:** 10.3389/fgene.2021.821535

**Published:** 2022-01-10

**Authors:** André F. Seixas, Ana P. Quendera, João P. Sousa, Alda F. Q. Silva, Cecília M. Arraiano, José M. Andrade

**Affiliations:** Instituto de Tecnologia Química e Biológica António Xavier, Universidade Nova de Lisboa, Oeiras, Portugal

**Keywords:** nucleotide modification, oxidative stress, 8-oxo-G, RNA oxidation, ROS, quality control of damaged RNA

## Abstract

Bacteria have to cope with oxidative stress caused by distinct Reactive Oxygen Species (ROS), derived not only from normal aerobic metabolism but also from oxidants present in their environments. The major ROS include superoxide O_2_^−^, hydrogen peroxide H_2_O_2_ and radical hydroxide HO^•^. To protect cells under oxidative stress, bacteria induce the expression of several genes, namely the SoxRS, OxyR and PerR regulons. Cells are able to tolerate a certain number of free radicals, but high levels of ROS result in the oxidation of several biomolecules. Strikingly, RNA is particularly susceptible to this common chemical damage. Oxidation of RNA causes the formation of strand breaks, elimination of bases or insertion of mutagenic lesions in the nucleobases. The most common modification is 8-hydroxyguanosine (8-oxo-G), an oxidized form of guanosine. The structure and function of virtually all RNA species (mRNA, rRNA, tRNA, sRNA) can be affected by RNA oxidation, leading to translational defects with harmful consequences for cell survival. However, bacteria have evolved RNA quality control pathways to eliminate oxidized RNA, involving RNA-binding proteins like the members of the MutT/Nudix family and the ribonuclease PNPase. Here we summarize the current knowledge on the bacterial stress response to RNA oxidation, namely we present the different ROS responsible for this chemical damage and describe the main strategies employed by bacteria to fight oxidative stress and control RNA damage.

## Introduction

### Oxidative Stress and Reactive Oxygen Species

**FIGURE 1:**
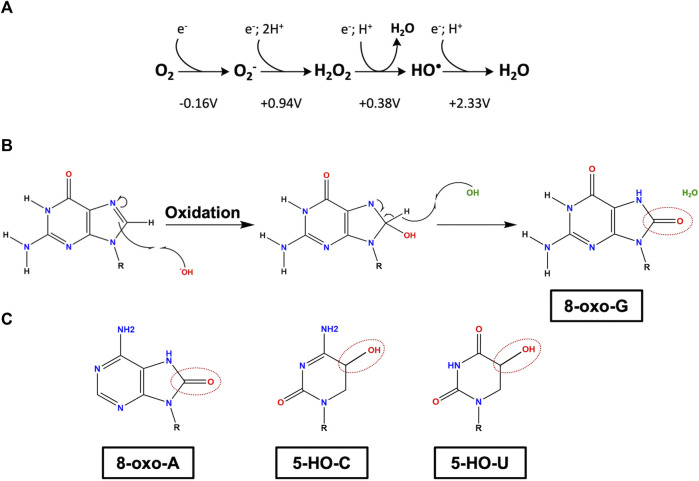
Main oxidative lesions found in RNA. **(A)** Redox state of molecular oxygen. From left to right: molecular oxygen (O_2_), superoxide anion (O_2_^−^), hydrogen peroxide (H_2_O_2_), hydroxyl radical (HO^•^) and water (H_2_O). The reduction potentials are shown regarding the standard concentration of O_2_ to be 1M at pH 7 (adapted from Imlay 2009). **(B)** Reaction of a guanosine with a hydroxyl radical, yielding 8-oxo-7,8-dihydroguanosine (8-oxo-G). **(C)** Three modified RNA nucleobases: oxidized adenosine 8-oxo-7,8-dihydroadenosine (8-oxo-A), oxidized cytosine 5-hydroxycytosine (5-HO-C) and oxidized uridine 5-hydroxyuridine (5-HO-U). Oxidation sites are marked as dashed circles. R represents the ribose group.

Oxygen is an abundant component of the earth’s atmosphere and a crucial player in many forms of life. However, oxygen comes with a risk that is the production of highly reactive oxygen species (ROS), which chemically modify by oxidation a variety of macromolecules (RNA, DNA, proteins, and lipids) (Markkanen 2017). Consequently, the structure and therefore the function of these macromolecules can be affected, usually resulting in cell toxicity. In bacteria and other organisms, ROS can be either generated intracellularly, as result of aerobic metabolism or exogenously from the outside environment, as consequence of local exposure to increased levels of oxidative agents (Dubbs and Mongkolsuk 2012; Fasnacht and Polacek 2021). Cells possess mechanisms to counteract oxidation and can tolerate low levels of ROS. Indeed, at low levels, ROS can act as signaling molecules controlling different cellular processes, such as quorum sensing, biofilm formation or bacterial self-destruction (Zhao and Drlica 2014; Dryden et al., 2017). However, when there is an imbalance between the amount of ROS and the ability to eliminate them, cells suffer oxidative stress (Berghoff and Klug 2012). Three naturally occurring ROS species, superoxide anion (O_2_−), hydrogen peroxide (H_2_O_2_), and hydroxyl radical (HO^•^), display different reactivities and are the ones with major relevance in aerobic environments and oxidative stress (Figure 1A).

### Superoxide Anion O_2_^−^

O_2_^−^ is a non-diffusible free radical formed when O_2_ removes one electron from a donor. It can be produced both intracellularly and exogenously (Kehrer 2000; Imlay 2009). Inside bacterial cells, the major source of O_2_^−^ is the autoxidation of enzymes like dehydrogenases, glutathione reductase and cytochromes P450 (Farr and Kogoma 1991; Gort and Imlay 1998). In eukaryotic cells, the most important source of ROS is the mitochondria *via* the electron transport chain system (Malla and Kwakye 2021). In phagocytes, O_2_^−^ is largely produced by NADPH oxidase and is used for destruction of pathogens (Shatwell and Segal 1996; Kong and Lin 2010). Exogenously O_2_^−^ can come from quinone-like compounds such as plumbagin and menadione or herbicidal paraquat (Farr et al., 1985; Suntres 2002; Criddle et al., 2006). Superoxide anion O_2_^−^ has a half-life in the range of microseconds and it oxidizes several targets like ascorbate and thiols (Kehrer 2000). Additionally, it can be dismutated to H_2_O_2_ and O_2_ through the action of superoxide dismutase (SOD) (Fridovich 1989) and act as a reducing agent of transition metals like Fe^3+^ (Farr and Kogoma 1991).

### Hydrogen Peroxide H_2_O_2_

Hydrogen peroxide is the simplest member of the class of peroxides. The main sources of internal H_2_O_2_ are the autoxidation of flavoenzymes and the dismutation of O_2_^−^ and contrary to superoxide anion, hydrogen peroxide can easily cross the cellular membrane (Imlay 2009). There are specific environments with residual amounts of oxygen that can be hubs of H_2_O_2_ accumulation, such as the anoxic interfaces in soil, hydrothermal vents (Tapley et al., 1999; Ogino et al., 2018) and the intestine epithelium (Espey 2013). One possible theory to explain the presence of H_2_O_2_ in the intestine is its production by lactic acid bacteria (LAB) (Imlay 2009). There is evidence that LAB metabolism can use oxygen and release H_2_O_2_ to their environment (Seki et al., 2004). Released H_2_O_2_ can also cause growth arrest of other bacterial species (Pericone et al., 2000; Tong et al., 2007; Bao et al., 2017). H_2_O_2_ is more stable when compared to O_2_^−^ with a half-life in the range of minutes (Kehrer 2000), and its decomposition is mediated by peroxidases like catalase (Winterbourn 2013). The most relevant reaction in which H_2_O_2_ is involved is undoubtedly the Fenton reaction (Fenton 1894). In this reaction, H_2_O_2_ interacts with metal cofactors such as iron (Fe^2+^) producing the highly reactive hydroxyl radicals HO^•^ (Shcherbik and Pestov 2019).

### Hydroxyl Radicals HO^•^

HO^•^ is one of the most dangerous ROS due to a half-life in the range of nanoseconds and its extreme reactivity given the high electron potential of +2.33 V (Figure 1A). It can attack most of the organic molecules in the vicinity of its formation, reacting either with proteins, lipids, or nucleic acids, especially with thiamine and guanosine (Farr and Kogoma 1991). HO^•^ can arise from absorption of a photon by tryptophan residues and from photochemical reactions in natural waters (Vaughan and Blough 1998). However, the most relevant source of HO^•^ in biological systems is the Fenton reaction since iron is such an important micronutrient (Liochev et al., 1999). Note that the ROS levels of the different species are interconnected. As mentioned above, O_2_^−^ reduces Fe^3+^ and its dismutation consequently produces H_2_O_2_; hence when the intracellular concentration of O_2_^−^ increases, the concentration of H_2_O_2_ and subsequently HO^•^ also increases. Contrarily to O_2_^−^, which can be eliminated by SOD, HO^•^ cannot be eliminated by an enzymatic reaction (Reiter et al., 1995).

### Reactive Oxygen Species and Cell Damage

An increase in ROS species is perilous to the cell as they cause an extensive damage to several targets, which include all biomolecules like proteins, lipids, and nucleic acids (DNA and RNA). As result of oxidation, the structure and function of these macromolecules are affected, with potentially toxic consequences that can ultimately result in cell death. Most protein oxidative modifications occur in the side chains of amino acids like oxidation of thiols and the formation of carbonyl groups affecting protein structure, conformation, and function (Dalle-Donne et al., 2006; Morzel et al., 2006; Zhang et al., 2013). Cysteine and methionine are the most susceptible amino acids to oxidation because they contain reactive sulfur atoms (Shacter 2000). In humans, most oxidized proteins are eliminated but some can accumulate and contribute to the damage associated with several diseases (Stadtman 2001). When ROS attack lipids, the products of this oxidation are lipid peroxides that mostly affect polyunsaturated fatty acids, which are critical components of cellular membranes (Niki 2009; Yin et al., 2011). Consequently, the accumulation of lipid peroxides results in changes in membrane permeability and fluidity (Dobretsov et al., 1977) and can affect ion channels, inactivate membrane transport proteins, disrupt homeostasis, and affect signaling pathways (Welsch 1987; Blair 2008; Su et al., 2019).

Nucleic acids are very susceptible to chemical damage given the reactivity of the nitrogen and oxygen atoms of the nucleobases (Simms and Zaher 2016). Among the four DNA nucleobases, guanine is the most prone to oxidation given its lower reduction potential (Steenken and Jovanovic 1997) and its oxidation can interfere with DNA metabolism like transcription and replication (Lee and Kang 2019). Contrarily to other macromolecules, damaged DNA cannot be replaced so it needs to be repaired to remain viable (Lee and Kang 2019). The main repair mechanism to correct non-bulky DNA lesions caused by oxidative stress is the base excision repair (Ignatov et al., 2017). Unlike DNA, no repair mechanisms have yet been identified for oxidized RNA, which imposes a challenge to the cell in order to keep homeostasis. This is even more important as RNA is more susceptible to oxidation than DNA (see below).

## RNA Oxidation

RNA accounts for 80–90% of the total nucleic acids (Hangauer et al., 2013; Li et al., 2020), and it affects the post-transcriptional regulation of gene expression (dos Santos et al., 2018; Quendera et al., 2020). ROS can attack RNA in different ways, whether it is through formation of abasic site, strand breaks or modifications of base and sugar moieties (Liu et al., 2012; Küpfer and Leumann 2014; Tanaka and Chock 2021). When compared to DNA, RNA molecules are more prone to oxidation for several reasons: 1) Being mostly single-stranded, the nucleobases of the RNA molecules are more exposed to ROS (Hofer et al., 2005); 2) RNA is less associated with proteins compared to DNA, making it more vulnerable (Cobley et al., 2018); 3) In physiological conditions the concentration of ribonucleotides is much higher than deoxyribonucleotides; therefore, the modifications are more likely to be incorporated into RNA than in DNA (Yan and Zaher 2019) and 4) RNA lacks repair mechanisms in contrast to what happens with DNA (Küpfer and Leumann 2014).

RNA molecules can be oxidized by HO^•^ derived from the Fenton reaction by detaching the hydrogen from the C-H bonds, resulting in several oxidation adducts, namely: 8-oxo-7,8- dihydroguanosine (8-oxo-G), 8-oxo-7,8-dihydroadenosine (8-oxo-A), 5-hydroxyuridine (5-HO-U) and 5-hydrocytosine (5-HO-C) (Simms and Zaher 2016; Yan and Zaher 2019) (Figures 1B,C). Although all four nucleobases are affected by ROS, the most common oxidation lesion found in cells is the 8-oxo-G given that guanosine is the base with the lowest reduction potential (Radak and Boldogh 2010; Shcherbik and Pestov 2019; Estevez et al., 2021). Under physiological conditions, the frequency of 8-oxo-G is approximately of 1 per 100.000 of unmodified guanosines (Shen et al., 2000). This modification has a high propensity to cause mutations in the open reading frame leading to changes in gene expression and the development of aberrant proteins (Ishii and Sekiguchi 2019; Yan and Zaher 2019). Furthermore, 8-oxo-G can dramatically reduce the rate of peptide-bond formation, strongly impairing protein synthesis (Thomas et al., 2019).

**FIGURE 2:**
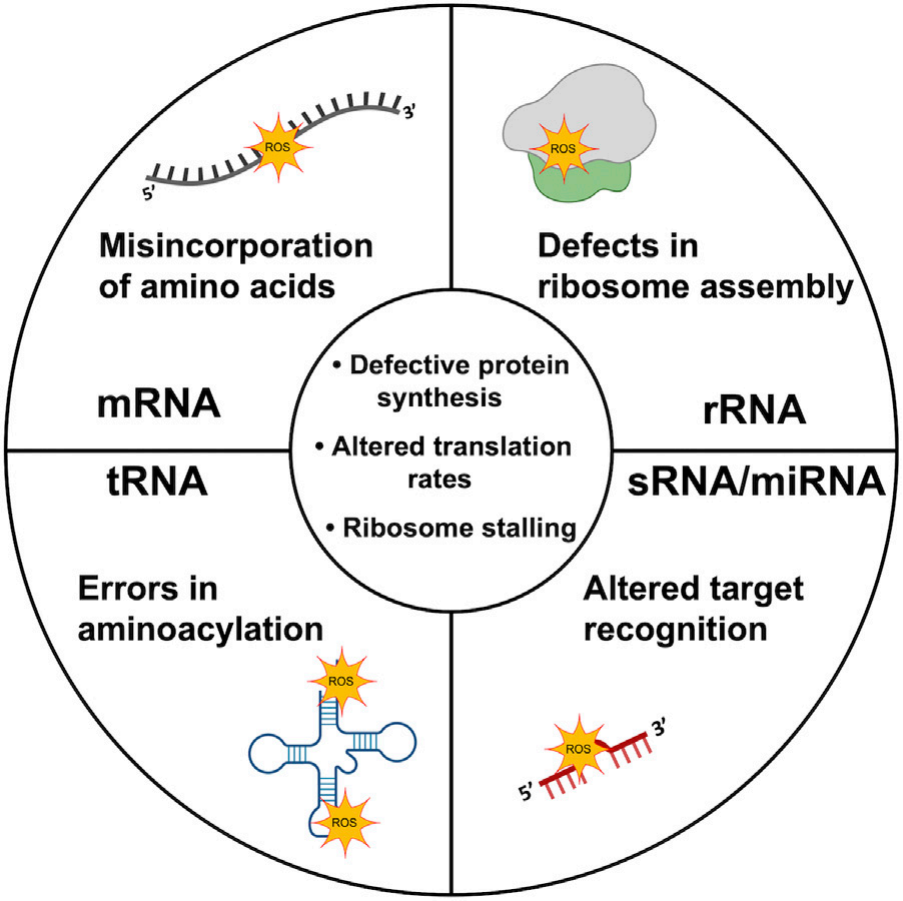
Oxidation of different RNA species. Virtually all RNA species are vulnerable to RNA oxidation. The scheme depicts major consequences of oxidation damage of rRNA, mRNA, tRNA and sRNA/miRNA molecules. In the middle of the scheme are represented defects common to oxidation of all RNA molecules. Molecule templates were retrieved from BioRender database.

When compared to DNA, the study of RNA damage by oxidation has been neglected, and this is mainly due to the assumption that RNAs tend to have a short half-life due to a rapid turnover before causing deleterious effects (Malla and Kwakye 2021; Tanaka and Chock 2021). This neglection resulted in an underestimation of the toxic effect of RNA oxidation on cells (Li et al., 2020). Next, we will provide examples on how RNA oxidation affects different RNA species within a cell (Figure 2).

### Ribosomal RNA

Ribosomal RNA composes the vast majority of total RNA molecules within a cell and is responsible for the structural and functional core of the ribosome (Cech 2000; Andrade et al., 2018; Dos Santos et al., 2020). Despite rRNAs being highly structured molecules, it is well established that rRNAs are a target for oxidation, not only in bacteria but also in higher organisms, and this has been associated with diseases (Ahmad et al., 2017; Willi et al., 2018). As we could expect, oxidation-induced modifications in the rRNA will affect important functional sites of the ribosome. In *Escherichia coli* it was shown that both the 16S rRNA (component of the small ribosomal subunit) and the 23S rRNA (component of the large ribosomal subunit) are subject to oxidation by H_2_O_2_ *in vivo*, although the 23S rRNA seems more prone to nucleobase oxidation (Willi et al., 2018). Specifically, it was shown that modification of three inner residues of the 23S rRNA (A2451, C2063, U2585) located in the catalytic center of the ribosome affects multiple steps of translation, such as peptide bond formation or tRNA binding to the ribosome. Oxidation of rRNA at specific positions within the ribosome can lead to defects in ribosome assembly (Simms and Zaher 2016). In addition, it was recently shown that the absence of methylations in the 23S rRNA at specific positions could make it more susceptible to oxidative stress, suggesting that oxidative damage to RNA can be regulated by the presence of post-transcriptional nucleoside modifications like methylation (Estevez et al., 2021).

### Transfer RNA

Transfer RNAs are structured molecules with great abundance and stability in the cell. These molecules decode the different codons on mRNA and allow the correct amino acid to be added to the nascent polypeptide. Certain post-transcriptional modifications normally found in tRNAs like the 5-methylaminomethyl-2-thiouridine (mnm5s2U) were also found to reduce oxidation in tRNAs (Estevez et al., 2021). Oxidation of the tRNA nucleobases is likely to induce a profound effect on the overall structure of the molecule, thus affecting protein synthesis. An *in vitro* study where liquid chromatography tandem mass spectrometry was applied to *E. coli* tRNA showed that upon oxidation induced by UV radiation, several tRNA nucleosides were modified with the 8-oxo-G adduct (Sun et al., 2018). Specifically, oxidative damage was mostly found to affect the anticodon and variable loop regions sequences of tRNA (Sun et al., 2020), which correlates with translational errors in the decoding process and aminoacylation (Yan and Zaher 2019). Zhong et al. (2015) showed that under oxidative stress caused by H_2_O_2,_ the vast majority of tRNAs in *E. coli* were decreased *in vivo*. This was shown to occur due to nonspecific extended tRNA degradation, in contrast to eukaryotic cells where tRNAs are cut in halves (Thompson et al., 2008). However, another report using a different *E. coli* strain revealed the reduction of only tRNA^Gly^ upon oxidation also with H_2_O_2_, with the global pool of tRNA remaining unchanged (Leiva et al., 2020). Therefore, the extent of tRNAs affected by oxidation is still debatable. The reduction of tRNA levels significantly slows down the translation elongation speed (Zhu and Dai 2019). This could be beneficial for bacteria as oxidative stress increases the probability of protein misfolding, which can be toxic to the cell. Concomitantly, overexpression of tRNAs was found to protect cells against oxidative stress, presumably by decreasing ribosome jamming found upon oxidation treatment, which causes the premature dissociation of RNA from ribosomes (Zhong et al., 2015).

### Messenger RNA

Messenger RNA is responsible for carrying the information encoded in DNA to synthesize proteins. The 8-oxo-G modification can affect the translation process in *E. coli* by interfering with the A site of mRNA codons leading to the incorporation of a different amino acid (Simms et al., 2014). Recent studies expanded the mechanism by which 8-oxo-G affects the decoding process. This adduct was found to induce a *syn* conformation of the mRNA molecule, causing it to pair preferentially with a tRNA carrying adenosine instead of cytidine, therefore, disrupting the translation rate (Thomas et al., 2019; Zhu and Dai 2019). From these studies it was also possible to conclude that the mRNA alterations caused by 8-oxo-G seem to affect mRNA-tRNA interactions in the ribosome decoding center. From the three main steps of the translation process, it is the elongation step that seems to be most affected by oxidative stress, with increasing levels of stalled ribosomes found on the mRNA molecule upon oxidation treatment, as observed in *E. coli* and cyanobacteria (Nishiyama et al., 2006; Zhong et al., 2015).

### Small Non-coding RNA

Small non-coding RNAs play an important role in the regulation of gene expression (Kavita et al., 2018; Quendera et al., 2020). In the literature there is no current evidence of direct oxidation of sRNAs in prokaryotes, although it seems likely that they are also targets for ROS, as it is the case of microRNAs (miRNAs) in eukaryotes. Namely, the microRNA miR-184 is highly oxidized and enriched in 8-oxo-G nucleotides upon H_2_O_2_ treatment (Wang et al., 2015). This oxidative modification influences the function of miR-184, which caused a substantial downregulation of Bcl-xL and Bcl-w mRNAs, promoting apoptosis to a higher extent. Recently, it was also reported that the miRNA miR-1 is preferentially oxidized in cardiac hypertrophy cases (Seok et al., 2020). Therefore, it is reasonable to think that like in miRNAs, bacterial sRNAs can also be oxidized under specific conditions. Further research is necessary to clarify whether this assumption proves to be true.

However, several studies demonstrated the importance of bacterial and even archaeal sRNAs in response to oxidative stress, namely by regulating the expression of important enzymes that protect the cell from ROS attacks. For instance, several sRNAs are known to regulate catalase expression in different bacteria. In *Deinococcus radiodurans*, the sRNA OsiA binds directly to the catalase *kat*A mRNA and stabilizes the transcript (Chen et al., 2019). In this bacterium it was also shown that in conditions of oxidative stress, another sRNA called OsiR is highly expressed and base-pairs with the mRNA of KatE2, resulting in increased transcript levels and increased translation of KatE2, a second catalase of this bacterium (Gao et al., 2020). In *Pseudomonas stutzeri* A1501, the NfiS sRNA was also found to bind to the mRNA of the catalase coding gene *kat*B (Zhang et al., 2019). Other sRNAs can regulate the expression of superoxide dismutase, as it the case of *Staphylococcus aureus* RscA which represses the translation of *sodA* mRNA and promotes the oxidative stress response via SodM (Lalaouna et al., 2019). Nevertheless, the role of sRNA is not restricted to regulation of ROS scavenging enzymes. sRNAs have been shown to act on multiple targets (Andrade et al., 2013) and may contribute for bacterial resistance to oxidative stress, namely through regulation of the expression of alternative transcription factors (Fröhlich and Gottesman 2018).

## Quality Control Pathways to Eliminate Oxidated RNA

Oxidized nucleobases incorporated into either DNA or RNA are potentially mutagenic and constitute a major challenge for cell survival. In bacteria, oxidized DNA bases (which block replication or originate mutations) can be removed by base excision repair enzymes (namely MutM, endonucleases IV and VIII) (for more information see Imlay 2013). When the oxidative damage is too profuse, SOS response is triggered, allowing RecA protein to repair DNA oxidation-induced lesions. RecA itself can also be oxidized, but this can be reversed by Msr proteins to maintain a level of reduced functional RecA necessary to carry out both efficient recombination and SOS system repair (Henry et al., 2021). In a striking contrast, no similar mechanisms of repair have yet been reported for oxidized RNA. Such pathways may exist but simply have not been recognized yet. In favor of this view, a mechanism of repair for alkylated RNA by oxidative demethylases has already been reported, proving that damages in RNA bases are repairable (Aas et al., 2003). However, two RNA-binding proteins, MutT and PNPase, have been identified to participate in surveillance mechanisms that recognize and degrade oxidized RNA.

### MutT/Nudix Family Members

Incorporation of oxidized ribonucleotides during RNA synthesis can lead to mutations (Li et al., 2014). *E. coli* RNA polymerase is ten-times less efficient in using 8-oxo-GTP than GTP (Taddei et al., 1997) and the human RNA polymerase II presents the same discriminatory activity (Hayakawa et al., 1999). MutT protein (a member of the Nudix family) was found to hydrolyze both 8-oxo-dGTP and 8-oxo-GTP, preventing their integration into DNA and RNA, respectively (Maki and Sekiguchi 1992; Taddei et al., 1997). In *Mycobacterium tuberculosis*, MutT1 and ADP-ribose pyrophosphatase (ADPRase) were shown to degrade 8-oxo-dGTP and 8-oxo-GTP/GDP (Patil et al., 2013). Such enzymatic activity is well conserved and human cells have four proteins responsible for degradation of oxidized nucleotides that are MutT homologues, namely MTH1, MTH2, MTH3 and NUDT5 (reviewed in Rudd et al., 2016).

### Polynucleotide Phosphorylase

Bacterial PNPase is widely conserved and a major 3′-5′ exoribonuclease involved in RNA degradation (dos Santos et al., 2018). In *E. coli*, PNPase was found to have high affinity for 8-oxo-G containing RNA when compared to undamaged RNA (Hayakawa et al., 2001). In agreement with this result, PNPase-deficient *E. coli* cells are more sensitive to hydrogen peroxide and other oxidants than the wild-type, and the levels of 8-oxo-G were found to strongly accumulate in the PNPase mutant strain, as result of deficient elimination of these defective RNA molecules. Complementation of the mutant corrects both the growth defect and reduces the levels of 8-oxo-G, suggesting a direct role of PNPase in controlling the oxidative damage (Wu et al., 2009). The importance of PNPase to fight oxidative stress has been extended to other bacterial species, such as *D. radiodurans* (Chen et al., 2007) and *Yersinia pestis* (Henry et al., 2012), where PNPase was required to protect cells under oxidative stress. Moreover, human PNPase (hPNPase) is located mainly in the mitochondria where oxidative damage is elevated, which presupposes a role for hPNPase in controlling the level of oxidized RNA (Chen et al., 2006). PNPase-knocked down HeLa cells under H_2_O_2_ treatment presented reduced growth and higher 8-oxo-G content than the control (Wu and Li 2008). Overall, PNPase is a major enzyme acting in the quality control of RNA and can discriminate among oxidized and non-oxidized forms of RNA, showing higher affinity to binding and degradation of oxidation modified RNAs in different organisms.

Another example of a protein that binds oxidized RNA is the mammalian Y box-binding protein 1 (YB-1), which specifically binds with high affinity to 8-oxo-G-containing RNA oligonucleotides (Hayakawa et al., 2002). The YB-1 domain responsible for RNA-binding is the cold shock domain and it has more than 40% homology to the major cold shock protein in *E. coli*, CspA (Matsumoto and Wolffe 1998). It is a multifunctional protein that can bind to both RNA and DNA and is important for transcriptional and translational regulation. When full length YB-1 was introduced and overexpressed in *E. coli*, bacteria became more resistant to paraquat oxidative stress (Hayakawa et al., 2002). However, when the RNA-binding domain was removed, cells were more susceptible to the oxidant. YB-1 could work as an RNA chaperone and be involved in the sequestration of oxidized RNA, preventing the translation of 8-oxo-G-containing mRNAs (Li et al., 2006). On the other hand, YB-1 could bind to oxidized ribonucleotides and recruit other factors for subsequent degradation. It is possible that both PNPase and YB-1 could have a cooperative function that protects cells against oxidized RNA, however the detailed mechanisms are still elusive (Ishii and Sekiguchi 2019).

## Scavenger Enzymes Against Free Radicals in Bacteria

Bacteria (and other organisms) are only able to tolerate a certain amount of non-harmful levels of ROS. To mitigate this threat, bacteria have evolved several mechanisms to fight oxidative stress and avoid the imbalance of ROS levels that lead to cell toxicity. From these, the presence of scavenging enzymes that consume ROS (superoxide dismutases, catalases and peroxidases) are critical in self-defense mechanisms against oxidative stress in bacteria.

### Superoxide Dismutase for Degradation of Superoxide

Superoxide dismutase is a family of enzymes widespread among bacteria, which act as a metalloenzyme that converts superoxide anion to hydrogen peroxide and molecular oxygen (Steinman 1988). *E. coli* has two cytoplasmic enzymes (Fe-SOD and Mn-SOD) and a periplasmic enzyme (Cu-Zn-SOD) (Imlay 2013). High titers of cytoplasmic SODs maintain steady-state superoxide levels at a sub-nanomolar concentration (Imlay and Fridovich 1991) in order to avoid toxicity by O_2_^−^. Being structurally and kinetically similar, the role of Mn-SOD and Fe-SOD is to ensure that SOD activity is present under a wide range of metal bioavailability. The two isozymes are coordinately regulated in response to iron levels: when iron is abundant, Fur (Ferric uptake regulator) inhibits Mn-SOD transcription while Fe-SOD is synthesized and activated by iron; when iron levels are reduced, Fur is deactivated and there is transcription of both Mn-SOD and the sRNA RyhB, which will repress Fe-SOD mRNA (Tardat and Touati 1991; Massé and Gottesman 2002). In anaerobic conditions, only Fe-SOD synthesis persists to prepare *E. coli* for subsequent aeration and consequent superoxide formation. Fe-SOD enzyme is favored by evolution in anaerobic habitats due to the biological availability of iron. Regarding periplasmic SODs, they protect bacteria from superoxide that leaks from respiratory chain components to the outer part of the cytoplasmic membrane. These SODs are under the control of the RNA polymerase sigma factor RpoS (also called σ^38^, Sigma S or KatF), and are strongly induced when aerobic cells enter stationary phase (Imlay 2009).

### Catalases and Peroxidases for Degradation of Hydrogen Peroxide

Catalases are common in most bacteria, with exceptions found in Gram-positive bacteria like streptococci, enterococci and leuconostocs (Mishra and Imlay 2012). *E. coli* has three main enzymes that can degrade hydrogen peroxide: alkyl hydroperoxide reductase (Ahp), catalase G (KatG) and catalase E (KatE) (Seaver and Imlay, 2001). Ahp is a two-component (AhpC-AhpF) thiol-based peroxidase that transfers electrons from NADH to H_2_O_2_, reducing it to water. KatG belongs to the catalase-peroxidase family and is only weakly expressed in exponential cells. However, the transcriptional regulator OxyR strongly induces both *ahp*CF and *kat*G when cells are stressed by exogenous H_2_O_2_ (see below). KatE is strongly expressed in stationary phase cells only, since it is induced by the RpoS system (Schellhorn and Hassan, 1988). These functionally redundant enzymes are used at different conditions: peroxidases are the primary scavenger at low H_2_O_2_ concentrations, whereas catalases are used at higher levels of H_2_O_2_ or when cells are starved (Imlay, 2013). Additionally, in the *E. coli* periplasm, cytochrome c peroxidase (Ccp) receives electrons from the respiratory chain and transfers them directly to H_2_O_2_ (Partridge et al., 2007). Ccp instead of eliminating H_2_O_2_ allows the cell to use it as a terminal oxidant to support respiration (Khademian and Imlay, 2017). This function is beneficial only when molecular oxygen is unavailable.

## Perceiving Redox Signals From Reactive Oxygen Species

To protect cells under oxidative stress, bacteria induce the expression of several genes, namely the SoxRS, OxyR and PerR systems. The activation of these multigene transcriptional pathways is critical to the bacterial response to oxidative stress and will be next presented.

### SoxRS System

Bacteria have evolved transcriptional regulators capable of perceiving redox signals from ROS, and consequently regulate enzymatic antioxidant systems (Lushchak and Storey 2021). The SoxRS regulon is a response mechanism to O_2_^−^ and to redox-cycling compounds, but not to H_2_O_2_ (Gu and Imlay 2011). This system is controlled by two proteins, which activate consecutively two stages of transcription (González-Flecha and Demple 2000). SoxR is a homodimer, each with an iron-sulfur cluster [2Fe-2S] center (Hidalgo et al., 1997) and whose activity depends on its oxidation state. Only the oxidized form ([2Fe-2S]^2+^) is active and able to induce transcription of *sox*S (Gaudu and Weiss 1996). SoxS is a second transcriptional activator that induces the transcription of more than 100 genes with functions in antioxidant defense, damage repair and maintenance of central metabolisms that allow the cell to survive under oxidative stress (Nunoshiba et al., 1992; Wu and Weiss 1992). Some of the genes regulated by SoxS are *sod*A, *nfo*, *zwf*, *fum*C, *acn*A, *fur* and *ntr*A (González-Flecha and Demple 2000; Touati 2000; Pomposiello et al., 2003; Blanchard et al., 2007). After oxidative stress, SoxR is again reduced and inactivated through reducing systems encoded by *rse*C and *rsx*ABCDGE operon. Therefore, these systems maintain SoxR in its reduced state, preventing activation of the SoxRS regulon (Koo et al., 2003). Also, when oxidative stress decreases, SoxS is rapidly degraded by proteolysis (Griffith et al., 2004). The transcription factor SoxR is widely distributed in Actinobacteria and Proteobacteria. However, while in enteric bacteria SoxR activates the expression of SoxS which in turn controls the expression of a diverse class of genes involved in the stress response, in nonenteric bacteria (like *Pseudomonas aeruginosa* or *Streptomyces coelicolor*) SoxS is absent, and SoxR directly activates a small sets of genes in response to redox-cycling agents (Chiang and Schellhorn 2012; Kobayashi et al., 2014; Mettert and Kiley 2015).

### OxyR System

The OxyR system is primarily responsible for recognizing and maintaining hydrogen peroxide levels in the cell (Åslund et al., 1999). OxyR is a LysR-type transcriptional regulator, widespread in most Gram-negative bacteria but it has also been reported in some Gram-positive bacteria, such as *S. aureus* and *S. coelicolor* (Morikawa et al., 2006; Oh et al., 2007). The transcriptional activity of OxyR activity depends on its redox state, as it can either act as activator if in its oxidized form or as repressor when in reducing conditions (Christman et al., 1989; Heo et al., 2010). OxyR acts as a tetramer and specifically binds to the 5’ promoter regions of target genes at a conserved sequence motif (Toledano et al., 1994). In *E. coli*, the OxyR regulon spans more than 20 different genes, involved in several molecular mechanisms of adaptive response to redox stress, such as H_2_O_2_ detoxification (*kat*E and *ahp*CF), heme biosynthesis (*hem*H), reductant supply (*grx*A, *gor*A, *trx*C), repression of iron import (*fur*, encoding Fur regulator of ferric ion uptake) and others (Zheng et al., 1999; Zheng et al., 2001a; Zheng et al., 2001b). Additionally, OxyR upregulates the expression of OxyS, a small regulatory RNA that directs peroxide stress response (González-Flecha and Demple 1999; Fröhlich and Gottesman 2018). Although OxyS does not directly affect the combat against excessive H_2_O_2_, this sRNA seems to play a role in protection from H_2_O_2_-induced mutagenesis (Altuvia et al., 1997). Additionally, depletion of OxyS has shown to result in considerably higher levels of H_2_O_2_ in E. *coli* (González-Flecha and Demple 1999).

Regulation by OxyR in *E. coli* begins by sensing the levels of H_2_O_2_ at a specific cysteine residue in the protein (C199). Under regular conditions, OxyR is present in its reduced form (Zheng et al., 1998). Increased levels of H_2_O_2_ result in the rapid oxidation of OxyR: the peroxide molecule reacts with the thiol group of C199, leading to interaction with the C208-SH to form an intramolecular disulphide bond, inducing conformational changes which alter the DNA binding properties of OxyR, allowing effective interaction with RNA polymerase (Lee et al., 2004). This molecular mechanism is then reversed through feedback regulation, since oxidized OxyR induces the expression of the *grx*A and *gor* genes, encoding glutaredoxin 1 and glutathione reductase, which act to reduce OxyR (Zheng et al., 1998). Although the OxyR system has been mostly studied in *E. coli*, different bacteria have evolved in the direction to adapt their particular OxyR regulon to better suit their environmental niches: they may display differences in the molecular mechanism of H_2_O_2_ regulation, the number of OxyR homologs or the type of genes present in their regulon (further reviewed in Sen and Imlay 2021).

### PerR System

Despite OxyR importance, the most prevalent system for preventing peroxide oxidative stress in Gram-positive bacteria is the PerR system. PerR belongs to the ferric uptake regulator (Fur) superfamily of metalloregulatory transcription factors, firstly identified in *Bacillus subtilis* where it is found to be the key regulator of the H_2_O_2_ response (Bsat et al., 1998). PerR has also been identified in some Gram-negative bacteria (van Vliet et al., 1999; Li et al., 2004), found primarily in association with OxyR (Wu et al., 2006). PerR is a transcriptional factor that acts as a dimer and in response to metal ions inside the cell through metal-catalyzed oxidation (Mongkolsuk and Helmann 2002). Each subunit contains a structural site that binds irreversibly to zinc (Zn^2+^) and a regulatory metal binding site (Jacquamet et al., 2009). In *B. subtilis*, PerR binds either to manganese (Mn^2+^) or to ferrous iron (Fe^2+^), which is preferred in most conditions (Sen and Imlay 2021). The metal-bound conformation of PerR binds to DNA and this binding occurs at a specific Per box (Fuangthong and Helmann 2003) located in the promoter region or downstream from it, acting then as a repressor (Dubbs and Mongkolsuk 2012).

Regulation by PerR is based on the oxidation of ferrous iron (Fe^2+^) into ferric iron (Fe^3+^) at the regulatory site. Under non-stress conditions, binding of the regulatory metal stabilizes the conformation of the PerR dimer to better interact with DNA, which leads to the repression of the PerR regulon (Jacquamet et al., 2009). When intracellular H_2_O_2_ level increase, excess peroxide causes the oxidation of the iron ions in the regulatory sites of PerR through the Fenton reaction (Lee and Helmann 2006). PerR then undergoes conformational changes that render it unable to interact with DNA (Imlay 2015), leading to the derepression of PerR regulated genes. Most of these genes are involved in peroxide stress metabolism and protection (*kat*, *ahp*CF operon, *mrg*A), but some participate in metal homeostasis (*hem*AXCDBL operon) and surfactant production (*srf*A) (Chen et al., 1995; Bsat et al., 1996, 1998; Hayashi et al., 2005). Similarly to OxyR, different bacteria may have evolved to use PerR in different ways to better adapt to their environmental niche but PerR homologs tend to normally regulate similar groups of genes.

## Conclusion

Aerobic organisms have developed over time the ability to maintain the levels of intracellular ROS within specific limits, to prevent ROS insults that can damage distinct classes of biomolecules. However, when self-defense mechanisms fail to protect the cell, ROS levels increase and as result of this imbalance, the cell faces oxidative stress. Due to its chemistry, RNA is highly susceptible to oxidation, which leads to the modification of nucleobases (namely, the formation of 8-oxo-G) causing RNA injury with potential mutagenic consequences. Since RNA is at the center of cellular regulatory pathways, the chemical damage of RNA triggered by ROS has a wide impact in gene expression. The importance of this impact is even stressed as RNA is highly expressed, comprising most nucleic acids within a cell, with the existence of many different classes of RNAs (mRNA, rRNA, tRNA and sRNA). Virtually, all these different RNA species are vulnerable to oxidation-induced damage, affecting translation at various levels. Not only the rate of protein synthesis is slowed but also the formation of aberrant proteins is potentiated, what may affect cell fitness and even cause cell death. The consequences of RNA damage by oxidation are widespread through all domains of life. Interestingly, in humans, the oxidation of RNA is implicated in several diseases, like cancer (Guo et al., 2020), diabetes (Cejvanovic et al., 2018), and neurodegenerative disorders (Fimognari 2015). Bacteria developed quality control mechanisms to specifically degrade oxidized RNA, in order to guarantee RNA fidelity. These surveillance mechanisms also rely on RNA-binding proteins that show higher affinity to bind oxidized RNA rather than non-damaged RNA. However, the number of proteins which were identified to participate in these mechanisms is rather small, with the best-known examples relying on PNPase and MutT. Nevertheless, it seems likely that other RNA-binding proteins participate in the surveillance of oxidized RNAs. Overall, the study of the bacterial response to oxidative stress and oxidation of RNA is an area that will certainly attract more attention in the years to come.
